# Perspectives for immunotherapy: which applications might achieve an HIV functional cure?

**DOI:** 10.18632/oncotarget.7793

**Published:** 2016-02-29

**Authors:** Vincent Vieillard, Shahin Gharakhanian, Olivier Lucar, Christine Katlama, Odile Launay, Brigitte Autran, Raphael Ho Tsong Fang, Joël Crouzet, Robert L. Murphy, Patrice Debré

**Affiliations:** ^1^ Sorbonne Universités, UPMC Université Paris 06, INSERM, CNRS, Centre d'Immunologie et des Maladies Infectieuses (CIMI-Paris), Paris, France; ^2^ InnaVirVax, Cambridge Innovation Center, Cambridge, MA, USA; ^3^ InnaVirVax, Génopole, Evry, France; ^4^ AP-HP, Hôpital Pitié-Salpêtrière, Service des Maladies Infectieuses et Tropicales, Paris, France; ^5^ Université Paris Descartes, INSERM, CIC 1417, AP-HP, Hôpital Cochin, Paris, France; ^6^ AP-HP, Hôpital Pitié-Salpêtrière, Département d'Immunologie, Paris, France; ^7^ Center for Global Health, Feinberg School of Medicine, Northwestern University, Chicago, IL, USA

**Keywords:** HIV, immunotherapy, functional cure

## Abstract

The major advances achieved in devising successful combined antiretroviral therapy (cART) have enabled the sustained control of HIV replication. However, this is associated with costly lifelong treatment, partial immune restoration, chronic inflammation and persistent viral reservoirs. In this context, new therapeutic strategies deserve investigation as adjuncts to cART so as to potentiate immune responses that are capable of completely containing HIV pathogenicity, particularly if cART is discontinued. This may seem a dauntingly high hurdle given the results to date. This review outlines the key research efforts that have recently resurrected immunotherapeutic options, and some of the approaches tested to date. These areas include promising cytokines or vaccine strategies, using different viral or non-viral vectors based on polyvalent “mosaic” antigens and highly conserved HIV envelope peptides, broadly neutralizing antibodies or new properties of antibodies to improve the control of immune system homeostasis. These novel immunotherapeutic strategies appear promising *per se*, or in combination with TLR-agonists in order to bypass the complexity of the interplay between immune activation, massive CD4^+^ T-cell loss and viral persistence.

## INTRODUCTION

Effective treatment with combined antiretroviral therapy (cART) has achieved impressive reductions in morbidity and mortality among patients infected with human immunodeficiency virus type 1 (HIV-1). Despite these advances, the success of cART is nevertheless frustrated by the need for costly lifelong treatment because it is impossible to completely eliminate the virus from patients, even when viral replication is suppressed at the periphery [1]. cART has also been associated with the development of viral resistance, and the increased risk of several non-AIDS disorders is now well documented, including that of cardiovascular, vascular, hepatic, renal, metabolic, neurological and some oncological diseases, more particularly in individuals who initiated cART at later stages of the infection [2]. This problem was recently demonstrated in a macaque model of SIV infection where even the initiation of treatment during the first three days post-infection was unable to prevent the constitution of a viral reservoir [3]. Furthermore, Lorenzo-Redondo et al showed very nicely that HIV can continue to replicate and replenish the viral reservoir despite potent cART [4]. New immunotherapeutic strategies therefore need to be explored, as an alternative or supplement to cART, targeting a cure and/or normalization of the immune status of treated patients.

The first approach concerns a “sterilizing cure” to enable complete eradication of the virus. The prototypical, and unique, example is the well-known “Berlin patient”, diagnosed with acute myeloid leukemia and then transplanted with CD34^+^ stem cells from a donor who was homozygous for CCR5 Δ32, and in whom viral replication remained absent, despite the discontinuation of cART [5]. Recently published follow-ups of this patient strongly suggest that a cure of HIV-1 has indeed been achieved [6]. However, hematopoietic stem cell transplantation is too risky, too complex and too costly for the majority of HIV-infected individuals worldwide, so it is likely that therapeutic strategies targeting eradication will not see widespread use in the near future. Strategies that induce latently infected cells to produce virus have recently generated considerable enthusiasm, including treatments with histone deacetylase (HDAC) inhibitors and other latency reversing agents; however, the clinical effects to date have been modest or not yet demonstrated [7–11].

The second approach is a “functional cure” without the complete eradication of HIV-1, associated with the development of effective host immunity to fight HIV-1 [12]. This is based on the strong immune responses observed in a very small proportion of untreated HIV-infected patients, namely long-term non-progressors and elite controllers, who are able to sustain high CD4^+^ T-cell counts and/or control HIV-1 replication below detectable levels [13, 14], and patients who experience long-term virological remission following the discontinuation of antiretroviral therapy that was initiated at an early stage [15].

## HOW IMMUNE RESTORATION CAN BE ACHIEVED IN THE HIV SETTING

**Figure 1:**
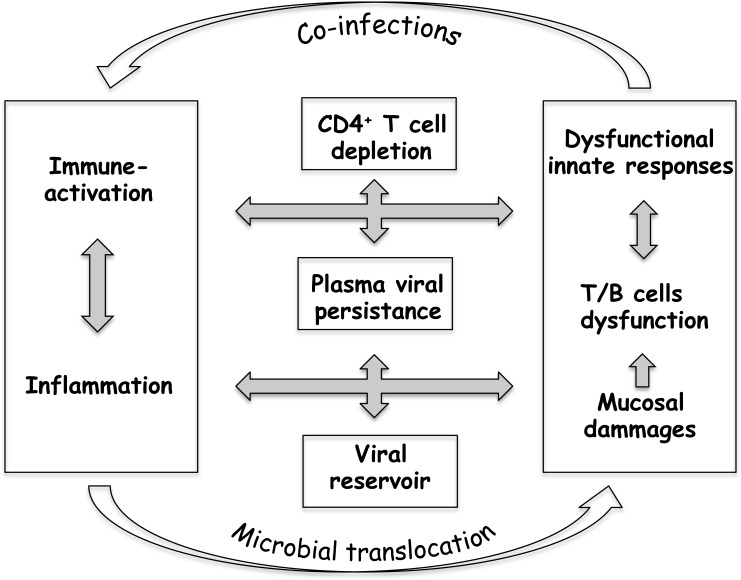
Poor immune restoration under cART sustains a “vicious circle” in HIV-1 infection

HIV-1 infection causes profound and often irreversible changes to the innate and adaptive immune systems. CD4^+^ T-cells are progressively depleted, whereas the CD8^+^ T-cell population expands, and much of the immune system is chronically activated. Even under cART, immune activation and chronic inflammation can persist for years, and certainly participate in the continuous replenishment of viral reservoirs, thus constituting an obstacle to an HIV cure [16–18]. The strongest evidence that HIV triggers immune activation has come from recent treatment intensification studies. The addition of raltegravir caused a concomitant reduction in the frequencies of both infected and activated cells [18–20]. The association between residual levels of immune activation and viral persistence after cART thus suggests that these two phenomena may be connected, which is in line with the recent findings of the START study [21]. These observations have increasingly focused clinical research on more robust endpoints that include preservation of the CD4^+^ T-cell count, associated with the control of immune activation and chronic inflammation (Figure 1).

To improve the effectiveness of treatments, a clearer understanding of the mechanisms underlying immune deterioration is necessary; without this knowledge, an efficacious clinical strategy will remain elusive. One of the most intriguing phenomena is the massive depletion of bystander non-infected CD4^+^ T-cells (Table 1). For example, several reports have shown that HIV-1 proteins, including gp120, Tat and Nef, can follow a variety of death pathways to initiate apoptosis in uninfected CD4^+^ T-cells [22–24]. An alternative proposal is that CD4^+^ T-cells may be killed by NK cells, and notably by the presence of stress molecules such as NKp44L, the cellular ligand of the activated natural killer (NK) NKp44 receptor [25]. In the context of HIV, this ligand is expressed specifically on bystander non-infected CD4^+^ T-cells [26, 27], whereas it is sequestrated by the HIV Nef protein within HIV-infected CD4^+^ T-cells. Taken together, these data may, at least partially, explain the preferential depletion of bystander non-infected CD4^+^ T-cells and development of the viral reservoir [28, 29]. It is interesting to note that this massive loss of non-infected CD4^+^ T-cells is associated with high levels of tissue inflammation and immune cell activation, which could increase both the number of potential HIV target cells susceptible to infection, and the homeostatic proliferation of latently infected cells [30, 31].

**Tabel 1:** Principal factors implicated in the HIV-induced loss of CD4^+^ T lymphocytes and immune functions, listed in alphabetical order

Antibody-dependent cellular toxicity and cytotoxic T-lymphocytes via the attachment of circulating gp120 to normal CD4^+^ T-cells: Anti-CD4^+^ T-cell action via auto-antibodies, Anti-CD4^+^ T-cell suppressive factors by CD8^+^ T-cells and/or NK cells. Apoptosis via immune activation. Cytokine cytotoxicity. Cytopathic effect of HIV on CD4^+^ T-cells and CD4 progenitors. Destruction of the bone marrow, lymph nodes architecture Immunosuppression by gp120, gp41, gag, Tat. Infiltrating malignancies affecting the bone marrow and other sites. Nutritional deficiencies. Opportunistic infections of the bone marrow. Permeability of the CD4^+^ T-cell membrane. Production of cytokines Selective destruction of memory CD4^+^ T-cells. Suppressive effect on immune complexes.

There are many reasons why the drastic reduction in viral load seen under cART does not result in normalization of the immune system; as well as the classic causes (CD4^+^ T-cell nadir, HIV RNA level, and host genetic factors), several other factors can be considered:

- Age; generally speaking, immune senescence may be both a cause and a consequence of immune activation, leading to a vicious circle [32].
- Co-infections; hepatitis C virus (HCV) co-infection results in a higher level of immune activation [33]. Likewise, cytomegalovirus has been identified as a cause of CD8^+^ T-cell activation in HIV-infected subjects under cART [34].
- Microbial translocation; the irreversible destruction by HIV of gut-associated lymphoid tissues (GALT), even after years of cART [35, 36]. This has been linked to the proportion of CD8^+^ T-cells over-expressing CD38 and type I interferons in the blood [37]. Microbial translocation may therefore be a driver of immune activation, and thereby of CD4^+^ T-cell loss [38]. This systemic activation of tissues in the gastrointestinal tract also contributes to the general increase in inflammation [17, 39, 40].

Furthermore, from a qualitative point of view, if CD4^+^ T-cell recovery under cART is based on the production of new CD4^+^ T-cells, then the T-cell repertoire may at least be partially restored. However, if the rise in the CD4 count results mainly from CD4^+^ T-cell proliferation and survival, the repertoire will remain uncompleted, even though the total number of CD4^+^ T-cells has normalized [41–43].

The complexity of the interplay between immune activation, massive CD4^+^ T-cell loss and viral persistence, as outlined in Figure 1, therefore suggests that important account needs to be taken of controlling immune system homeostasis when developing novel immunotherapy strategies to combat AIDS.

## IMMUNOTHERAPY IN THE DISEASE SETTING

### Objectives

Immunotherapy should aim to elicit immune restoration, because the immune responses work in concert to control HIV disease. The great majority of past and current strategies have focused on the viral component, but a key question has arisen: is it possible to generate HIV-specific immune responses capable of completely containing viral replication, even in tissue sanctuaries, when cART is discontinued? During randomized trials, no strategy has yet demonstrated its lasting and potent effectiveness in terms of controlling viral load, or delaying clinical progression [44]. This review focuses mainly on immunotherapy to control pathogenicity, prevent immune activation by HIV-1 and reduce inflammation, thereby normalizing the immune system, as an alternative strategy designed to promote directly effective and specific immune responses. It is likely that the efficacy of therapeutic interventions will depend on the disease stage at the initiation of antiretroviral therapy [45]. Different approaches are being tested, including cytokine therapies, therapeutic vaccines, and antibody therapies with different molecular targets.

### What about cytokine therapy?

Cytokines play a pivotal role in modulating immune responses. Both agonist and antagonist therapies directed against cytokines, cytokine receptors or signaling pathways are currently the subject of intense investigation. Treatments based on interleukin (IL)-2, IL-7 IL-15, and IL-21, which all utilize the common CD132 γ-chain in their receptor complexes, seem to be able to improve the CD4^+^ T-cell count. A trial involving intermittent IL-2 therapy in cART-naive patients was able to demonstrate sustained increases in CD4^+^ T-cell numbers that enabled the deferral of cART [46]. However, larger international randomized trials designed to assess the effects of IL-2 in HIV-1-infected individuals with cART-induced viral suppression revealed no evidence of an improvement in HIV-1 immunity [47], with IL-2 promoting the expansion of FOXP3^+^ regulatory T-cell populations [48]. These disappointing findings, as well as the toxicity associated with IL-2 therapy, thus prompted research on the immunomodulating properties of other cytokines such as IL-7 and IL-15 in terms of improving T-cell homeostasis. A dose escalation trial in cART-treated HIV-infected patients showed that IL-7 was well-tolerated and could improve the available TCR repertoire, potentiate cytotoxic T-cell activity and restore CD4^+^ T-cell homeostasis [49, 50]. On the other hand, supplementation with IL-15 promoted the differentiation of central memory CD4^+^ T-cells into shorter-lived transitional or effector memory T CD4^+^, which are crucial to immune control [51, 52]. Finally, IL-21 improved T-cell cytotoxicity as well as enhancing antibody production and not increasing viral load in chronically untreated SIV-infected macaques. In particular, higher levels of intestinal Th17 cells were associated with lower rates of intestinal T-cell proliferation, microbial translocation and systemic activation/inflammation [53].

To date, these cytokine-based agents are of uncertain benefit in HIV-infected patients; indeed, high levels of certain cytokines may participate in tissue inflammation and immune cell activation, which in turn could increase the number of potential HIV target cells susceptible to infection. Recently, Herasimtschuk *et al.* [54] reported a randomized immunotherapeutic study in cART-treated HIV^+^ individuals designed to investigate the effects of combining IL-2 with therapeutic immunization using a clade B DNA vaccine; their preliminary data suggest that this strategy may improve the CD4^+^ T-cell count, restore anti-HIV-1 responses and reduce immune activation. Therefore, carefully administered at the correct time, cytokines might perhaps be combined with other strategies to achieve a clinical benefit.

### What about therapeutic vaccination?

**Figure 2:**
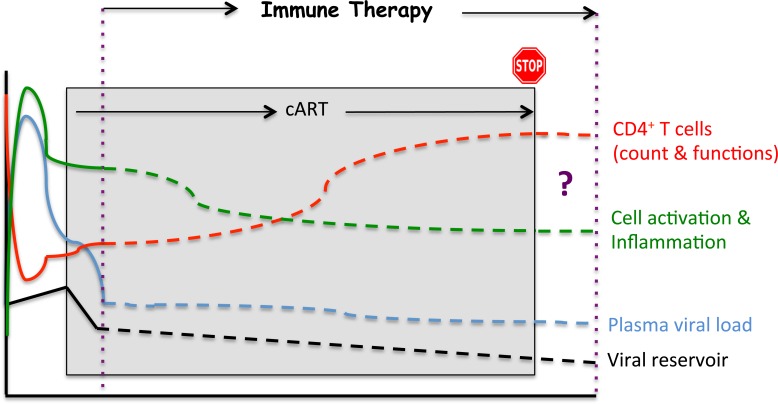
Potential impacts of an immunotherapeutic strategy

Although no product has yet reached the stage of a phase III clinical trial, a growing number of candidate vaccines are being evaluated in phase I/II or phase II trials conducted in treatment-naive and/or cART-treated patients [55–65] (Table 2), as recently reviewed by Barouch *et al.* [66]. Some of these viral and non-viral vectors induce HIV-antigen-specific CD8^+^ T-cell responses, associated in some instances with HIV-specific B-cell responses. Some of these vaccines have also achieved modest effects on viral load in HIV-infected patients [67]. This argues in favor of improving the vectors and/or antigens. In addition, a combination of such vaccines with other immunotherapeutic approaches could act in synergy (see below). Other strategies are based on HIV peptides; they benefit from a relatively simple design which allows for a thorough characterization of the immune responses induced and avoidance of the complications that have been associated with complex viral vaccine preparations. To date, peptide vaccines to protect CD4^+^ T-cells have received relatively little attention in terms of vaccine design, although these cells are the primary targets of HIV infection and protecting them will inevitably interfere with this infection; such an effect could contribute to protection against the disease, mainly by controlling immune-mediated inflammation and cell activation (Figure 2) [68–70].

**Table 2:** The principal therapeutic vaccines

Vaccine	Additional description	Phase	Trial Registry Identifier*	Reference
vCP1452	ALVAC-based vaccine	2	NCT00056797	
MRK Ad5 HIV-1 gag	Adenovirus-based vaccine	2	NCT00080106	[56, 57]
ISS T-002	HIV Tat-based vaccine	2	NCT00751595	[58, 59]
GTU-multiHIV B + LIPO-5	DNA + lipopeptide vaccines	2	NCT01492985	
DermaVir LC002	DNA-based vaccine	1/2	NCT00270205	[60–62]
Synthetic vaccine	HIV Tat-based vaccine	1/2	NCT01793818	
THV01	Lentiviral-based vaccine	1/2	NCT02054286	
DNA-GTU	Plasmid DNA-based vaccine	1/2	NCT02457689	
AGS-004	Patient-derived dendritic cells + HIV antigens	1/2	NCT01069809	
Epimmune	DNA-based vaccine	1	NCT00052182	[64]
rMVA-HIV (env/gag [TBC-M358] + tat/rev/nef [TBC-M335])	Vaccina Ankara-based vaccine	1	NCT00107549	[65]
rFPV-HIV (env/gag [TBC-F357] + tat/rev/nef [TBC-F349])	Fowlpox-based vaccine	1	NCT00107549	[65]
MVA.HIVconsv	MVA-based vaccine	1	NCT01024842	
MAG pDNA + rSVIN HIV-Gag	DNA + VSV-based vaccine	1	NCT01266616	
HIVAX	Lentiviral-based vaccine	1	NCT01428596	
ChAdV63.HIVconsv + MVA.HIVconsv	Adenovirus + MVA-based vaccines	1	NCT01712425	
iHIVARNA-01	TriMix + HIV antigen naked messenger RNA	1	NCT02413645	
D-GPE DNA + M-GPE MVA	DNA and MVA viral vector vaccines	1	NCT01881581	

Subdominant peptide epitopes from HIV-1 restricted to common HLA have been used in combination with an adjuvant to vaccinate treatment-naive HIV-1-infected individuals. New HIV-1 specific CD4^+^ and CD8^+^ T-cell responses were induced in all patients; however, there were no significant changes to HIV-1 viral load or the CD4^+^ T-cell count [71]. This highlights the importance of selecting optimum peptides for vaccination, because of the problem of HIV-1 diversity. This challenge could be resolved using inert mosaic sequences created by computer algorithms to maximize the coverage of potential epitopes from worldwide strains [72]. Studies in macaques have shown that mosaic sequences can enhance T-cell responses, but they have not yet been evaluated in humans, and the immune response they will elicit remains unknown [73]. In an attempt to address the issue of HIV-1 diversity, highly conserved motifs can also be used to elicit immune responses capable of recognizing viruses from multiple clades.

**Figure 3:**
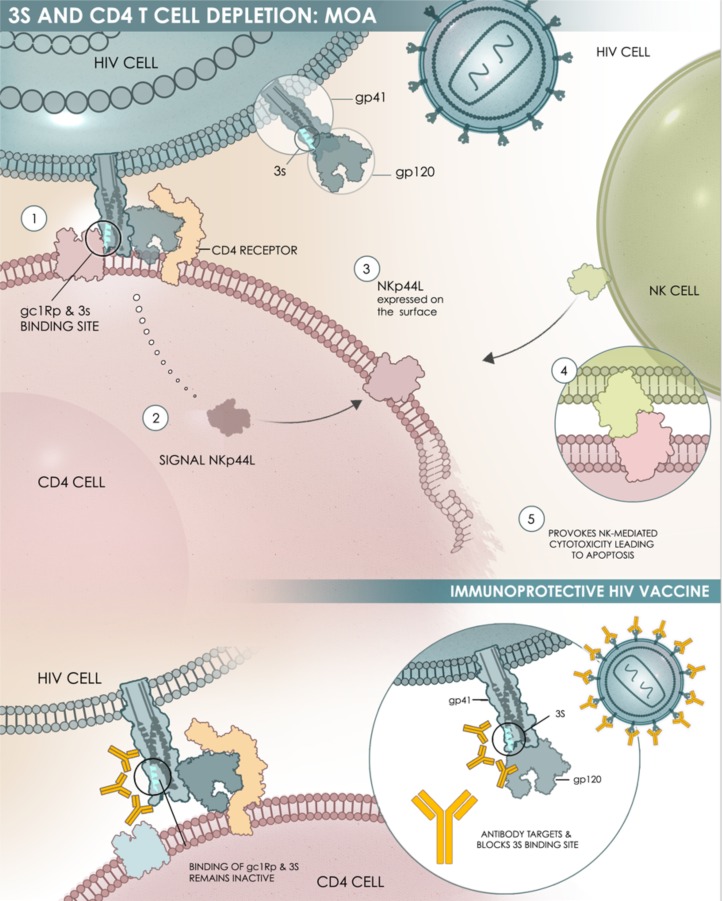
Schematic representation of the mechanism of action of 3S The 3S motif of the gp41 envelope protein of HIV-1 acts through a subsequent cascade of interactions: Steps 1 & 2: The 3S motif binds to its specific receptor (gC1qR) on CD4^+^ T-cells (CD4); Step 3: This interaction induces a molecular pathway that leads to NKp44L translocation at the surface of CD4^+^ T-cells; Steps 4 & 5: the interaction between NKp44L and its receptor, with NKp44 expressed on activated NK cells, induces NK-mediated cytotoxicity and then CD4^+^ T-cell depletion.

Here, we briefly review the most representative peptide vaccine approaches tested to date:

- A C4-V3 polyvalent peptide vaccine comprising four peptides which contain T-helper epitopes from four constant regions of gp120, all HLA-B7-restricted cytotoxic T-lymphocytes and B-cell neutralizing epitopes from the gp120 variable region (V3) of four clade B isolates. A pilot Phase I study (DATRI-010) in 10 HIV-infected patients revealed both immunogenicity and safety in the patients, with no modulation of the CD4^+^ T-cell count or plasma HIV RNA levels [74].
- Vacc-4x is based on four synthetic peptides corresponding to HLA-A2-restricted conserved domains of the HIV-1 protein p24. A phase II randomized, double-blind, placebo-controlled trial was performed in 136 HIV-infected patients on cART who were randomly assigned to receive Vacc-4x. After the discontinuation of cART, a modest but significant reduction in viral load was noted, but the changes to CD4^+^ T-cell counts were not significant, although proliferative responses were induced in both CD4^+^ and CD8^+^ T-cell populations [75].
- VAC-3S is a peptide vaccine based on the highly specific and conserved 3S motif localized in a gp41 HIV-1 region, exposed to the surface of the envelope protein (Pancera & Kwong, NIAID, USA - Personal communication). This motif interacts with the gC1qR, a receptor for the globular form of the C1q complement factor, to induce both a decline of the CD4^+^ T-cell count and an increase in viral load [26, 76]. In simian HIV (SHIV)-infected macaques immunized with the 3S-derived peptide, the decline of the CD4^+^ T-cell count was prevented in both peripheral and secondary lymphoid tissues [77, 78]. Alongside these observations, we highlighted an inhibition of immune inflammation (production of C-reactive protein, and TNF-α) (personal data), and the activation and proliferation of CD4^+^ T-cells, linked to a significant protection of central memory CD4^+^ T-cells [78, 79]. Overall, these data revealed a mechanism of action for the 3S motif composed of successive steps, which are outlined in Figure 3. Blocking this pathway in HIV-infected patients could protect CD4^+^ T-cells and decrease immune activation and inflammation, thereby achieving immune protection and restoring immune homeostasis. A First-In-Human study performed in 33 HIV-infected patients under cART revealed that VAC-3S is immunogenic and safe [80]. Significant changes from baseline in VAC-3S vaccine responders were observed (increase in the percentage of CD4^+^ T-cells and in the CD4^+^/CD8^+^ T-cell ratio, reduction of total proviral DNA) indicating that this therapeutic vaccine approach is of interest (manuscript in preparation) [81, 82]. An international phase II, randomized, double-blind, placebo-controlled study is currently evaluating the safety, tolerability and immunogenicity of three different VAC-3S immunization schemes in 90 virologically-controlled HIV-1 infected patients with CD4^+^ T-cell counts of between 200 and 500 cells/mm^3^ [*ClinicalTrials.gov identifier NCT02041247*] [83].

The studies performed to date have demonstrated that safe and effective vaccines based on a highly conserved motif are possible; they can protect CD4^+^ T-cells and have made important con­tributions to our understanding of the path towards developing such a vaccine with immune homeostatic properties.

### What about antibody therapy?

Antibodies (Abs) are used to either enhance immune responses or inhibit negative regulatory pathways. The ultimate goal is to reduce inflammation, prevent immune activation by HIV-1 and promote immune responses. This can be illustrated in three different situations, which reflect some putative new concepts:

- The therapeutic blocking of PD-1 and CTLA-4: Programmed cell death 1 (PD1) and cytotoxic T-lymphocyte antigen 4 (CTLA4) are over-expressed in CD4^+^ T-cells, and more particularly in the memory cells of all infected subjects, except elite controllers. The administration of monoclonal Abs (mAbs) to block PD-1 during chronic SIV infection in macaques prolonged survival; it also reduced immune over-activation and the expression of type 1 IFN stimulated genes, and enhanced immunity to gut-associated pathogens [84, 85]. Similarly, the administration of an anti-PD-1 ligand promoted a lower rebound viral load in infected macaques [86].
  The results obtained with anti-CTLA-4 mAbs in macaques were unfortunately less encouraging, with an increased activation of CD4^+^ T-cells and viral replication at mucosal sites [87].
- The lesson of RV144: in this vaccine, which is able to confer protection of 30% against HIV-1 acquisition [88], Env-directed IgA Abs can block the IgG-mediated antibody-dependent cellular cytotoxicity (ADCC) effector function [89, 90]. While the underlying mechanism remains unclear, non-neutralizing Abs may offer protection through effector functions such as ADCC, which occur in conjunction with cells of the innate immune system [91]. While much work remains, these new results represent potentially crucial clues to better understanding the protective function of Abs in the context of HIV-1.
- The role of HIV-1 broadly neutralizing Abs: our understanding of what constitutes a broadly neutralizing antibody against HIV has recently been revolutionized by the isolation of extremely broad and potent neutralizing mAb from HIV-infected individuals [92–96]. Few of them are being studied in man regarding the protective therapeutic benefit of passive immunization [97–100]. While their infusions are well-tolerated, transient decreases of viral load have been observed, in line with the pharmacokinetics of the antibodies [99, 100]. One issue concerns the emergence of resistant HIV-1 viruses, so it is necessary to either combine broadly neutralizing mAbs together or with other immunotherapies.
- The role of anti-3S Abs: These are inversely associated with the rate of CD4^+^ T-cell count decrease, although they do not neutralize the virus [101]. Importantly, patients with high levels of anti-3S Abs experience a significantly delayed progression to CD4^+^ T-cell levels below 200 cells/mm^3^, and a very significantly lower level of cellular viral DNA [102]. Taken together, these data have led us to propose a possible application for anti-3S Abs as a “functional cure” [103]. Although much work still needs to be done, these new results offer potentially important clues to a clearer understanding the function of Abs during HIV-1 progression.

### What about other immunotherapies?

The strategies described above that are used to block various immune pathways need to be investigated in more depth, as does their potential synergy with other clinical approaches. In particular, in order to achieve a more efficient HIV cure, immunotherapy strategies could be combined with new types of drug such as the toll-like receptor 7 (TLR7) agonist, which can induce a transient activation of CD8^+^ T-cells (and of CD4^+^ and NK cells) and reduce the amount of viral DNA within cells from HIV-positive donors on cART [104], or by using a TLR9 agonist capable of reducing the viral reservoir in HIV-infected patients and enhancing HIV-specific CD8^+^ T-cell immunity [105].

## CONCLUSIONS

The current clinical and therapeutic picture of HIV infection argues strongly in favor of new strategies that could promote restoration of the immune defenses. We hypothesize that an immunotherapeutic strategy enabling the re-establishment of T-cell homeostasis in HIV-infected patients is necessary to permit a sustainable peripheral and central control of viral burst in the context of a “functional cure”. Indeed, immunotherapeutic approaches that can directly enhance effector functions against HIV and/or HIV-infected cells could be combined, and their expected synergy with immunotherapies would result in immune restoration. At all events, future progress will depend on an iterative relationship between novel findings from preclinical studies, including the impact in innate and other immune effector participants beyond conventional CD4^+^ and CD8^+^ T-cells [90], and the proposal of combined, multiple immune-based therapeutic strategies to fight HIV disease and enable patients to be off therapy for long periods of time.
